# Understanding physical exercise among individuals with substance use disorders using an integrated theoretical perspective of the health action process approach and theory of planned behavior

**DOI:** 10.3389/fpsyg.2024.1377430

**Published:** 2024-04-10

**Authors:** Yong Meng, Ting Zhu, Wei Chen, Hongjie Zhou, Lanping Tao, Xiaoteng Wang, Mengya Li, Xiaofang Zhang, Dongshi Wang, Xingyue Wu, Shaochen Luo, Cheng Hu

**Affiliations:** ^1^School of Social and Public Administration, East China University of Science and Technology, Shanghai, China; ^2^Legal Department, Zhejiang Drug Rehabilitation Administration, Hangzhou, China; ^3^Mental Health and Guidance Center, Ningbo University, Ningbo, China; ^4^School of Education, Tianjin University, Tianjin, China; ^5^Faculty of Sports Science, Ningbo University, Ningbo, Zhejiang, China; ^6^Shiliping Compulsory Isolated Detoxification Center, Quzhou, China

**Keywords:** substance use disorders, physical exercise behavior, health action process approach, theory of planned behavior, behavioral intention, self-efficacy, planning

## Abstract

**Introduction:**

Physical exercise is considered a useful non-pharmacological adjunctive treatment for promoting recovery from substance use disorders (SUD). However, adherence to physical exercise treatments is low, and little is known about what factors are associated with the initiation and maintenance of physical exercise behaviors. The aim of this study was to explore the psychosocial factors underlying these behaviors in individuals with SUD using an integrated theoretical model based on the health action process approach (HAPA) and the theory of planned behavior (TPB).

**Methods:**

A total of 1,197 individuals with SUDs (aged 37.20 ± 8.62 years) were recruited from 10 compulsory isolation drug rehabilitation centers in Zhejiang Province via convenience sampling according to a set of inclusion criteria. Self-reported data were collected to assess task self-efficacy (TSE), maintenance self-efficacy (MSE), recovery self-efficacy (RSE), outcome expectations (OE), action planning (AP), coping planning (CP), social support (SS), subjective norms (SN), attitude behavior (AB), behavioral intention (BI), perceived behavioral control (PBC), risk perception (RP), exercise stage, and exercise behavior in this integrated model. ANOVA and structural equation modeling (SEM) were used to evaluate this model.

**Results:**

One-way ANOVA revealed that the majority of the moderating variables were significantly different in the exercise phase. Further SEM showed that the model fit the data and revealed several important relationships. TSE, RP, SS, AB, and SN were indirectly associated with physical exercise behavior in individuals with SUD through the BI in the SUD initiation stage. In addition, PBC was directly related to physical exercise behavior in individuals with SUD. In the maintenance stage, MSE, AP, CP and exercise behavior were significantly related. Moreover, AP and CP were mediators of BI and MSE.

**Conclusion:**

This study is the first attempt to integrate patterns of physical exercise behavior in individuals with SUD. The HAPA-TPB integration model provides a useful framework for identifying determinants of physical exercise behavioral intentions and behaviors in individuals with SUD and for explaining and predicting the initiation and maintenance of physical exercise behaviors in these individuals. Moreover, the model provides scientific guidance for the enhancement of physical exercise adherence in individuals with SUD.

## 1 Introduction

Substance use disorders (SUDs), including disorders involving the use of illicit drugs, alcohol, cannabis, or nicotine, have become an important, costly, and intractable global public health problems. The World Drug Report in 2023, published by the United Nations Office on Drugs and Crime (UNODC), states that more than 296 million individuals used drugs in 2021 worldwide, representing a 23% increase over the previous decade; in addition, the number of individuals suffering from SUDs has surged to 39.5 million, a 45% increase in the past decade (Vreugdenhi et al., 2012). Substance abuse not only destroys the health of people with SUDs but also leads to serious social problems that jeopardize the health of others and social stability.

Scholars have explored ways to reduce the risk of relapse from multiple perspectives, with physical exercise being recognized as a potential non-pharmacological adjunctive intervention to reduce the risk of relapse (Wang et al., 2014; Huang et al., 2019; Thompson et al., 2020). In the past decade, research on the topic of physical exercise and SUDs has been popular and has yielded promising theoretical achievements. Recent studies have reported the positive benefits of physical exercise, including promoting physical fitness (Xu et al., 2022), reducing cravings (Zhou et al., 2021), improving mood states (Rawson et al., 2015), and enhancing cognitive functions (Wang et al., 2015), in individuals with SUD. Moreover, the concept that the risk of relapse can be reduced by physical exercise has been implemented in drug rehabilitation practice. In China, a pilot program was started by 24 drug rehabilitation institutions in 2018, and by 2020, 226 compulsory isolation drug rehabilitation centers were established to carry out physical exercise treatment programs, enabling a total of more than 80,000 individuals with SUDs to participate in physical exercise (Feng et al., 2021).

However, outstanding achievements have been made in both theoretical explorations and practical applications regarding the rehabilitative benefits of physical exercise for individuals with SUD. However, the challenging issue of adherence to SUD participation in physical exercise looms large. In one of our 12-week aerobic exercise intervention studies, the rate of adherence of individuals with methamphetamine use disorders to a moderate-intensity aerobic exercise program was only 80.64% (Wang et al., 2017). In another of our studies, individuals with SUDs were asked to participate in 12 weeks of a group-based aerobic exercise program, and the adherence rate was only 82.93% (Zhu et al., 2022). Although the participants involved in these two studies were in compulsory isolation and received organized, supervised, and regular physical exercise, they still had low adherence rates. Moreover, even higher dropout rates have also been observed in physical exercise intervention studies for other SUDs. One study reported that 95 individuals with alcohol use disorders (AUDs) were randomized to participate in a 12-week aerobic or yoga program (at least 3 times per week), and only 49% of the participants completed the supervised exercise program (Welford et al., 2023). Other studies have reported that the prevalence of exercise adherence among people with AUDs is between 50 and 70% (Giesen et al., 2015; Thompson et al., 2020). Although physical exercise is known to have beneficial effects on brain plasticity and cognitive functioning improvements in people with SUDs (Wang et al., 2017, 2020), maintaining a regular physical exercise program is extremely challenging for individuals with SUDs, especially in the early stages of withdrawal. A lower adherence rate is associated with a greater risk of relapse, which can seriously undermine the rehabilitative benefits of physical exercise programs for individuals with SUD. Therefore, the topic of physical exercise adherence in individuals with SUD is a major concern that must be addressed not only for researchers but also for policymakers.

The participation of individuals with SUD in physical exercise is an example of the reshaping of health behavior, a process that can be initiated and maintained by multiple influencing factors. Moderate and major levels of addiction, higher body mass index, and lower educational attainment may be important factors in decreasing adherence to physical exercise participation in individuals with AUDs (Welford et al., 2023). Additionally, individuals with SUD show low compliance and may have difficulty maintaining physical exercise programs due to impairments in cognition and mental health as a result of chronic substance abuse (Abrantes and Blevins, 2019). Furthermore, individuals with SUD may lack sufficient motivation to engage in physical exercise, especially in the early stages of withdrawal (Abrantes et al., 2011). While previous studies have begun to focus on the factors that influence physical exercise adherence in individuals with SUD, they have not systematically identified the factors that influence individuals’ initiation and maintenance of physical exercise behaviors, especially psychosocial correlates (e.g., intentions). Few theoretical studies have addressed the initiation and maintenance of physical exercise behaviors in individuals with SUD, but attempts have been made to explain the psychosocial factors that underlie adherence to physical exercise among individuals in other groups from different theoretical perspectives, such as the transtheoretical model (Marshall and Biddle, 2001), self-determination theory (Standage and Ryan, 2020), theory of planned behavior (TPB) (Downs and Hausenblas, 2005) and the health action process approach (HAPA) (Barg et al., 2012; Shen et al., 2012). Of these theories, the TPB and the HAPA are considered more suitable and consistent than the other theories.

The TPB is one of the most commonly used social cognitive theoretical models for predicting health behavior change. The model theorizes that behavioral intention is the most direct factor influencing health behaviors and that it is determined by an individual’s behavioral attitudes, subjective norms, and perceived behavioral control; in addition, perceived behavioral control is thought to have a predictive role in the emergence of behaviors (Ajzen, 1985). Behavioral attitudes are individuals’ comprehensive assessments of a behavior based on their perception of the outcome of the benefits of the behavior, and positive behavioral attitudes enhance their willingness to participate in the behavior. However, subjective norms focus on normative views formed by significant others’ supportive attitudes toward one’s participation in a particular behavior. In addition, perceived behavioral control represents an individual’s evaluation of the required resources and barriers related to participating in the behavior. Due to its simplicity and ease of implementation, the TPB model has been recognized by numerous scholars in the field of exercise behavior change (Gomes et al., 2018; Gourlan et al., 2019; Ruiz et al., 2021; Hagger and Hamilton, 2023). The theory emphasizes the motivational role of attitudes and embodies the role of the objective environment in the two factors of subjective norms and perceived behavioral control. The TBP bridges the gap between the individual and the environment, transforms the constraints imposed on the individual by factors such as objective social environments and material conditions to the individual’s subjective perception, and explains the mechanism of the role of objective environmental factors on behavior. However, the theory has some limitations in explaining the crucial factors of behavioral change, and nearly 50% of the variance in behavioral intention and behavior is unexplained (Sheeran, 2002; Mceachan et al., 2011). In other words, individuals’ intentions to choose a new behavior do not lead to actual behavior change, and there is a gap between intentions and behaviors. As a result, the theory is commonly applied to explain and predict behavior. This limitation may be attributed to the fact that the theory is a static theoretical model that fails to adequately account for the cognitive variables involved in the dynamic sequential process of physical exercise behavior (Shen et al., 2010).

Unlike the TPB, the HAPA, which is typical of the stages model of exercise behavior, suggests that people go through multiple stages of health behavior change: before the decision stage (before action and non-intenders), after the decision stage and before the action stage (indictors), and during the action stage (actors). The HAPA suggests that the factors affecting individuals at different stages also differ (Schwarzer, 2008; Schwarzer and Hamilton, 2020). The before-decision stage of action results in behavioral intention, which is determined by risk perception, outcome expectation, and self-efficacy and is also called the motivation stage. In the after-decision and before-action stages, planning plays a crucial role in the realization of health target behaviors [average effect size of 0.65 (Gollwitzer and Sheeran, 2006)]; thus, it is also called the planning stage. Planning consists of two dimensions: action planning, which details when, where and how to perform the behavior (if-condition), and coping planning, in which possible obstacles to the goal are anticipated and ways of overcoming them are identified (then-condition). In this phase, where concrete action planning must be developed to lead to actual behavior, perceptual self-efficacy continues to play an influential role in motivating individuals to achieve goals by planning and attempting to take action, while the perception of risk loses its facilitating role. In the action phase (consisting of the initiation and maintenance phases), engagement and maintenance of the action are regulated solely by self-efficacy, with barriers and available resources (e.g., social support) determining the maintenance, withdrawal, and resumption of the behavior (Schwarzer and Hamilton, 2020). The HAPA also incorporates behavioral intentions and action planning as proximal predictors of true action into the structure of the model, thus forming a continuum of behavioral change encompassing intentions, planning, and true action (Schwarzer, 2008; Schwarzer and Hamilton, 2020). Since its formulation, the HAPA has been widely used in studies related to rehabilitation exercise in patients, and it is considered to provide a useful theoretical framework for evaluating physical exercise intentions and behaviors in the groups involved (Zhang et al., 2019; Schwarzer and Hamilton, 2020; Godoy-Izquierdo et al., 2023). The HAPA has also been used in numerous research studies on patients’ physical exercise. Similarly, the HAPA has several limitations; for example, studies have shown that risk perception does not explain the variance of behavior and intention well (Schwarzer et al., 2007; Kaczynski et al., 2008; Zhang et al., 2022), which means that risk perception is a negligible variable because most exercisers do not necessarily engage in or withdraw from exercise because they perceive the presence of risk. Another limitation is that the HAPA does not include social factors that influence exercise behavior or behavioral intentions (Chow and Mullan, 2010).

Given that the existing theories themselves have some shortcomings, some scholars have begun to try to combine multiple theories to construct a comprehensive theory of exercise behavior. Combining social cognitive theories (e.g., the TPB) and stage models of exercise behavior (e.g., the HAPA) to explore study exercise behavior has become a new approach. The HAPA indicates the role of variables mediating the relationship between behavioral intention and behavior, including maintenance self-efficacy and recovery self-efficacy, whereas the TPB fails to address the gap between intention and behavior. Thus, the HAPA has the potential to compensate for this deficiency in the TPB. The construction of an integrated HAPA-TPB model could improve the prediction of exercise behavior and clarify the specific roles of each moderating variable. Several researchers have integrated and revised the TPB and HAPA in the context of Chinese culture (Shen et al., 2010) and have been able to better explain the relationships among the variables involved in the various stages of physical exercise behavior among Chinese people (Zhou, 2014; Zhang et al., 2022).

The TBP is widely used to predict behavioral intentions for addiction treatment in individuals with SUD and is considered an effective screening tool (Savvidou et al., 2012; Zemore and Ajzen, 2014; Moeini et al., 2017; Bonny-Noach et al., 2023). In one such study, Savvidou et al. (2012) used an expanded version of the TPB to examine the social-cognitive predictors of the behavioral intentions of individuals with SUD in physical exercise treatment and found that attitudes and perceived behavioral control were strong predictors of physical exercise intentions. Although this study provided a theoretical framework for factors influencing physical exercise behavior in individuals with SUD, it did not systematically reveal how behavioral intentions influence physical exercise behavior in individuals with SUD due to limitations of the TPB itself. Therefore, the aim of the present study was to determine the factors associated with the initiation and maintenance of physical exercise behavior in individuals with SUD using an integrated HAPA-TPB model. We combined the relevant research and understanding in the field of exercise behavior and proposed the following main hypotheses: (1) the integrated HAPA-TPB model better predicts exercise behavior in individuals with SUD; (2) there are specificities in the characteristics of exercise behavior in individuals with SUD according to the exercise stage; (3) risk perception, outcome expectation, social support, behavioral attitudes, subjective norms, and action self-efficacy predict intention; and (4) in the integrated HAPA-TPB model, behavioral intention mediates the role of recovery self-efficacy, action planning, and coping planning in maintaining physical exercise behavior.

## 2 Methodology

### 2.1 Participants and procedure

A total of 1,235 individuals with SUD were recruited from 10 compulsory isolation drug rehabilitation centers in Zhejiang Province, China, to participate in the survey according to the following inclusion criteria and using a convenience sampling strategy. The inclusion criteria for individuals with SUD were as follows: (1) aged 18 years or older, (2) serving a compulsory isolation time of more than 6 months, and (3) had used drugs in the last 3 months and had used drugs for more than 1 year. Our survey was conducted in a face-to-face format; participants were informed of the aims and objectives of the study and provided with an anonymous, paper-based self-report questionnaire to complete. After missing values were eliminated, data from a total of 1,197 individuals with SUD were ultimately included in the statistical analysis. This study was approved by the Faculty of Sport Science Ethics Review Board (No. TY2022024), Ningbo University, and adhered to the Declaration of Helsinki.

### 2.2 Measures

The self-report questionnaire consisted of three parts: background information, a questionnaire related to the HAPA, and a questionnaire related to the TBP.

The background information survey mainly included questions on demographic variables such as age, gender, ethnicity and socioeconomic status, as well as substance abuse. The evaluation of the degree of substance addiction was derived from the relevant diagnostic criteria in the *Diagnostic and Statistical Manual of Mental Disorders* (Fifth Edition) (DSM-V).

The physical activity levels of the participants were assessed using the Chinese version of the *Physical Activity Rating Scale-3* (PARS-3) revised by Liang (1994). The PARS-3 examines an individual who is exercising in terms of intensity, frequency, and duration of physical activity. Each aspect is categorized into five levels, with intensity and frequency scored 1–5 on a scale of 1–5, and time scored 0–4, respectively. The exercise score = exercise intensity × exercise time × exercise frequency, and the range of the exercise score was 0–100 points. The retest reliability of the PARS-3 was 0.82 (Liang, 1994).

The stage of exercise is identified by the *Stages of Exercise Diagnostic Scale* (Richert et al., 2011). The scale categorizes exercise stages into three stages by using five items: the unintentional stage, intentional stage and action stage. Items such as “Please think back to the past 4 weeks, did you perform physical activity at least at moderate intensity for 30 min 3 times a week?” Participants were asked to choose one of three options to answer based on the actual situation.

An the *Exercise Self-Efficacy Scale* was used to measure task self-efficacy, maintenance self-efficacy, and recovery self-efficacy (Renner and Schwarzer, 2005). The task self-efficacy subscale consisted of 4 items (Cronbach’s α = 0.8650), items such as “Please select the level of certainty that you would be able to start participating in regular exercise in each of the following situations.” The maintenance self-efficacy subscale consisted of 11 items (Cronbach’s α = 0.848), such as “Please select the level of certainty you have that you will be able to maintain regular exercise in each situation.” The recovery self-efficacy subscale consisted of 4 items (Cronbach’s α = 0.7741), such as “Please select the level of confidence that you would still be able to restart regular exercise in each case.” All 11 of these items are scored on a 5-point Likert scale.

The *Expectation of Outcome Scale* was used to assess positive and negative outcome expectations (Renner and Schwarzer, 2005). Positive outcome expectancy was assessed with 9 items (Cronbach’s α = 0.8857), and the negative outcome expectancy subscale consisted of 3 items (Cronbach’s α = 0.5636). Items such as “Please select the confidence level you have in the advantages and disadvantages of participating in regular exercise in each situation” have options that are rated on a 5-point Likert scale.

*Risk perception* was measured by five items (Cronbach’s α = 0.8818), such as “If I continue to live as I do now, then my risk of developing diabetes will be high” (Renner and Schwarzer, 2005).

Action planning and coping planning were assessed through the *Planning Scale* (Renner and Schwarzer, 2005). The action planning subscale consisted of 5 items (Cronbach’s α = 0.8832), such as “For exercise I’m sure I have got a specific plan in place about when I’m going to start exercise.” And the coping planning subscale consisted of 4 items (Cronbach’s α = 0.8830), such as “For exercise I’m sure I have got a specific plan regarding what exercise obstacles I encounter and how I’m going to deal with them.” All 9 of these items are scored on a 5-point Likert scale. The Behavioral Intentions Scale consisted of 3 items (Cronbach’s α = 0.8393), such as “For me, over the next 4 weeks, I’m going to do at least 3 times a week, 20 min or more of physical activity each time” (Fishbein and Ajzen, 2010; Shen et al., 2012). All of these items are scored on a 7-point Likert scale.

The *Subjective Norms Scale* consists of 3 items (Cronbach’s α = 0.8474), such as “The people who are important to me endorse that I get at least 20 min of physical activity at least 3 times a week,” and is scored on a 6-point Likert scale (Fishbein and Ajzen, 2010).

The *Perceived Behavioral Control Scale* consists of 3 items (Cronbach’s α = 0.6457), such as “Do I have the ability to control my physical activity for 20 min or more at least 3 times a week for the next 4 weeks?,” and is scored on a 6-point Likert scale (Fishbein and Ajzen, 2010).

The *Behavioral Intention Scale* consists of 3 items (Cronbach’s α = 0.8393), such as “Over the next 4 weeks, I plan to do at least 3 physical workouts per week of 20 min or more each,” and is scored on a 6-point Likert scale (Fishbein and Ajzen, 2010; Conner, 2020).

The *Exercise Social Support Scale* consists of 5 items (Cronbach’s α = 0.8255), such as “Friends or family members have offered or said they would exercise with me in the past 3 months,” and is scored on a 5-point Likert scale (Hankonen et al., 2010).

These scales used in this study included the Exercise Self-Efficacy Scale, the Expectation of Outcome Scale, the Perceived Risk Scale, the Behavioral Intentions Scale and the Exercise Social Support Scale, all of which are Chinese localized and revised versions (Shen et al., 2012). The revised Chinese versions of the scales reflect the content measured, and their theoretical concepts are basically in line with those of the original scales; moreover, the revised versions of these scales are widely used in China (Shen et al., 2012; Zhou, 2014; Liu et al., 2015). In addition, the Chinese versions of the scales related to the TBP, including Behavioral Attitudes, Subjective Norms, Perceived Behavioral Control, and Intentions, were revised by Hu and Mao (2008), and the internal consistency coefficients of each scale ranged from 0.72 to 0.81, with good measurement equivalence and better compliance with all dimensions of the TBP.

Based on previous reports of communication with individuals with SUD, we adjusted the sources of social support in the Exercise Social Support Scale to “parents, relatives, friends, rehabilitation partners and rehabilitation staff” and changed “friends” to “rehabilitation partners and rehabilitation staff” in the subjective normative scale to better fit the social context of rehabilitation and physical exercise in which individuals with SUD live.

### 2.3 Statistical analysis

We evaluated the variability in the characteristics of participants with different addiction type, as well as in the exercise behavior-moderating variables between exercise phases by ANOVA or Chi-square tests with SPSS 28.0. The hypotheses were tested by structural equation modeling in AMOS 24.0. Cronbach’s α (>0.7), composite reliability (CR) (>0.7), and average variance extracted (AVE) (>0.5) were used to evaluate the consistency of the data in the measurement model. To assess the overall model fit, χ2/df, RMSEA, NFI, CFI, and IFI were selected as the model fit indicators. Among them, a *χ^2^/df* < 5 and RMSEA <0.08 were needed, with a value closer to 0 indicating a better model fit; an NFI, CFI, and IFI > 0.9 were required, with a value closer to 1 indicating a better model fit (Urbach and Ahlemann, 2010). Finally, we performed mediation effect tests using the bootstrap method (set up for 5,000 iterations) to obtain 95% confidence intervals for the parameter estimates. *p-*values <0.05 were considered to indicate statistical significance, and all tests were bilateral.

## 3 Results

### 3.1 Participant information

Information on the relevant characteristics of the participants included in the statistical analysis is described in Table 1. Furthermore, there were significant differences between the three different categories of individuals with SUD in terms of gender, ethnicity, socioeconomic status index, and substance abuse status (all *p* < 0.01).

**Table 1:** Demographic characteristics and substance abuse status of the participants included in the study (*n*/*M* ± *SD*).

	Stimulant use disorders	Heroin use disorders	Polydrug use disorders	*F*/*x*^2^	*p-*value	Total
Number	820	149	228			1,197
Age (years)	35.92 ± 8.44	40.04 ± 8.31	39.93 ± 8.40	29.97	0.00	37.20 ± 8.62
Gender						
Male	748 (91.20%)	147 (98.70%)	218 (95.60%)	13.68	0.00	1,113 (93.00%)
Female	72 (8.80%)	2 (1.30%)	10 (4.40%)			84 (7.00%)
Ethnicity						
Han	739 (90.10%)	112 (75.20%)	184 (80.70%)	32.09	0.00	1,035 (86.50%)
Minority	81 (9.90%)	37 (24.80%)	44 (19.30%)			162 (13.50%)
Socioeconomic Status	10.99 ± 2.73	9.54 ± 2.46	10.53 ± 2.76	18.81	0.00	10.72 ± 2.74
Marriage						
Single	307 (37.40%)	24 (34.20%)	68 (29.8%)	18.48	0.102	426 (35.60%)
Married	242 (29.50%)	48 (32.20%)	64 (28.10%)			354 (29.60%)
Divorce	226 (27.6%)	41 (27.50%)	78 (34.20%)			345 (28.80%)
Widowed	3 (0.40%)	2 (1.30%)	3 (1.30%)			8 (0.70%)
Cohabite	19 (2.30%)	4 (2.70%)	7 (3.10%)			30 (2.50%)
Separate	2 (0.20%)	1 (0.70%)	4 (1.80%)			7 (0.60%)
No report	21 (2.60%)	2 (1.30%)	4 (1.80%)			27 (2.30%)
Education						
Illiteracy	42 (5.10%)	22 (14.80%)	16 (7.00%)	54.96	0.00	80 (6.70%)
Elementary	171 (20.90%)	59 (39.60%)	59 (25.90%)			289 (24.10%)
Junior	415 (50.60%)	55 (36.90%)	104 (45.60%)			574 (48.00%)
Senior	139 (17.00%)	10 (6.70%)	38 (16.70%)			187 (15.60%)
College and above	53 (6.50%)	3 (2.00%)	11 (4.90%)			67 (5.60%)
Occupation						
Farm Laborer	147 (17.9%)	50 (33.60%)	60 (26.30%)	40.70	0.00	257 (21.50%)
Self-Employed	256 (31.20%)	25 (16.80%)	53 (23.20%)			334 (27.90%)
Manual Worker	180 (22.00%)	44 (29.50%)	49 (21.50%)			273 (22.80%)
Office-bearer	26 (3.20%)	4 (2.60%)	5 (2.20%)			35 (2.90%)
Technical Staff	22 (2.70%)	0 (0.00%)	6 (2.60%)			28 (2.30%)
General Staff	116 (14.20%)	13 (8.82%)	30 (13.10%)			159 (13.30%)
Not Employed	73 (8.90%)	13 (8.70%)	25 (11.00%)			111 (9.30%)
Income (1000yuan/month)	5.64 ± 5.37	3.96 ± 3.31	5.22 ± 5.61	6.69	0.02	5.35 ± 5.23
Drug abuse situation						
Smoking (year)	18.30 ± 8.52	21.91 ± 8.21	22.66 ± 8.24	30.36	0.00	19.58 ± 8.63
Drinking (year)	15.41 ± 9.65	18.14 ± 10.38	17.40 ± 11.15	6.94	0.00	16.13 ± 10.09
Drug use (year)	7.66 ± 5.12	11.01 ± 7.63	13.85 ± 7.21	102.24	0.00	9.26 ± 6.48
Abstinence (month)	13.58 ± 5.24	13.89 ± 5.13	13.33 ± 5.37	0.50	0.61	13.57 ± 5.25
Addiction Score	5.69 ± 3.78	6.76 ± 4.08	8.05 ± 4.02	34.80	0.00	6.27 ± 3.97

### 3.2 Analysis of exercise behavior stages

To further examine the extent to which the moderating variables of the integrated model matched across the different exercise behavioral stages, ANOVA and *post hoc* tests were conducted (Table 2). The results showed that all variables were significantly different (all *p* < 0.01) by exercise stage except for two variables, maintenance self-efficacy and risk perception. *Post hoc* tests revealed significant differences between the before-decision stage and the after-decision before-action stage (all *p* < 0.05), except for the moderating variables of maintenance self-efficacy, recovery self-efficacy, negative outcome expectation, and risk perception. Moreover, except for the maintenance self-efficacy and risk perception variables, significant differences (all *p* < 0.05) were observed between the before-decision and action stages of the after-decision before-action stage. These results suggest the existence of discontinuous features in different stages of exercise behavior.

**Table 2:** Summary of ANOVA and *post hoc* test results for moderating variables at each stage (*M* ± *SD*).

	Before Action stage (BD) (*n* = 395)	After decision and before action stage (AD/BA) (*n* = 135)	Action stage (A) (*n* = 667)	*F*-value	Turkey HSD	
					BD-AD/BA	AD/BA-A
Task self-efficacy	11.79 ± 3.92	12.96 ± 3.68	15.00 ± 4.06	83.39^***^	−1.17^**^	−2.03^***^
Maintenance self-efficacy	27.95 ± 8.19	29.22 ± 7.94	28.98 ± 10.34	1.73	−1.27	0.25
Recovery self-efficacy	7.37 ± 3.12	7.92 ± 2.79	6.92 ± 3.67	5.68^**^	−0.55	1.00^**^
Positive outcome expectations	29.84 ± 9.12	33.00 ± 8.61	35.54 ± 8.06	56.04^***^	−3.16^***^	−2.54^**^
Negative outcome expectations	8.90 ± 2.89	9.48 ± 2.83	10.24 ± 3.12	24.95^***^	−0.58	−0.76^*^
Action planning	13.04 ± 5.03	15.60 ± 5.24	17.49 ± 5.45	88.09^***^	−2.56^***^	−1.90^***^
Coping planning	9.83 ± 3.79	11.99 ± 3.86	12.96 ± 4.52	68.09^***^	−2.16^***^	−0.96^*^
Social support	11.92 ± 4.88	14.46 ± 4.89	16.44 ± 5.67	89.08^***^	−2.54^*^	−1.98^***^
Subjective norm	14.61 ± 5.25	16.17 ± 4.39	17.86 ± 4.05	65.11^***^	−1.56^**^	−1.69^***^
Attitude behavior	24.43 ± 8.86	28.53 ± 6.86	30.53 ± 6.36	86.02^***^	−4.10^***^	−2.00^*^
Behavioral intention	14.65 ± 5.33	17.06 ± 4.23	18.38 ± 4.79	71.58^***^	−2.41^***^	−1.32^*^
Perceived behavioral control	9.72 ± 3.55	10.82 ± 3.04	12.02 ± 3.95	47.98^***^	−1.11^**^	−1.20^**^
Risk perception	21.39 ± 8.61	21.76 ± 9.08	20.89 ± 9.28	0.72	−0.37	0.87
Exercise behavior	9.36 ± 12.26	14.18 ± 13.09	18.57 ± 18.93	39.36^***^	−4.82^**^	−4.39^*^

^*^p < 0.05, ^**^p < 0.01, ^***^p < 0.001.

### 3.3 Measurement model

The means, standard deviations, Cronbach’s values and bivariate correlations for all the model variables are presented in Table 3. The Cronbach’s alpha coefficients in each dimension ranged from 0.59 to 0.96, the CR coefficients ranged from 0.70 to 0.93, and the AVE coefficients ranged from 0.50 to 0.82. The results indicate that the measurement model has overall good reliability and convergent validity.

**Table 3:** Summary of means, standard deviations, Cronbach’s α, and bivariate correlation coefficients of the HAPA-TPB composite model variables.

	Mean	SD	Cronbach’s α	CR	AVE	TSE	MSE	RSE	POE	NOE	AP	CP	SS	SN	AB	BI	PBC	RP	EB
Task self-efficacy (TSE)	13.71	4.24	0.88	0.88	0.65	1													
Maintenance self-efficacy (MSE)	28.67	9.43	0.88	0.87	0.51	0.18^***^	1												
Recovery self-efficacy (RSE)	7.18	3.42	0.82	0.83	0.61	0.09^**^	0.59^***^	1											
Positive outcome expectations (POE)	33.37	8.87	0.93	0.93	0.57	0.60^***^	0.20^***^	0.11^***^	1										
Negative outcome expectations (NOE)	9.71	3.07	0.69	0.70	0.50	0.44^***^	0.36^***^	0.23^***^	0.69^***^	1									
Action planning (AP)	15.81	5.66	0.93	0.93	0.74	0.44^***^	0.26^***^	0.20^***^	0.44^***^	0.29^***^	1								
Coping planning (CP)	11.82	4.52	0.92	0.92	0.73	0.41^***^	0.33^***^	0.30^***^	0.40^***^	0.33^***^	0.72^***^	1							
Social support (SS)	14.73	5.72	0.90	0.90	0.66	0.30^***^	0.15^***^	0.089^**^	0.33^***^	0.25^***^	0.40^***^	0.37^***^	1						
Subjective norm (SN)	16.60	4.75	0.91	0.91	0.77	0.47^***^	0.03	0.00	0.47^***^	0.25^***^	0.34^***^	0.30^***^	0.34^***^	1					
Attitude behavior (AB)	28.29	7.83	0.96	0.96	0.82	0.52^***^	0.03	−0.01	0.51^***^	0.25^***^	0.38^***^	0.32^***^	0.34^***^	0.76^***^	1				
Behavioral intention (BI)	17.00	5.20	0.79	0.82	0.61	0.43^***^	0.01	−0.02	0.39^***^	0.18^***^	0.35^***^	0.31^***^	0.30^***^	0.73^***^	0.72^***^	1			
Perceived behavioral control (PBC)	11.13	3.87	0.59	0.70	0.53	0.36^***^	0.02	0.01	0.33^***^	0.15^***^	0.26^***^	0.20^***^	0.24^***^	0.59^***^	0.60^***^	0.56^***^	1		
Risk perception (RP)	21.16	9.04	0.92	0.92	0.53	0.025	0.27^***^	0.20^***^	0.05	0.13^***^	0.04	0.08^**^	0.09^**^	−0.07^*^	−0.06^*^	−0.08^**^	−0.08^**^	1	
Exercise behavior (EB)	15.03	16.91	0.72	0.81	0.57	0.26^***^	−0.02	−0.03	0.26^***^	0.16^***^	0.28^***^	0.27^***^	0.16^***^	0.24^***^	0.26^***^	0.21^***^	0.17^***^	−0.12^***^	1

^*^p < 0.05, ^**^p < 0.01, ^***^p < 0.001.

### 3.4 Structural model

**Figure 1:**
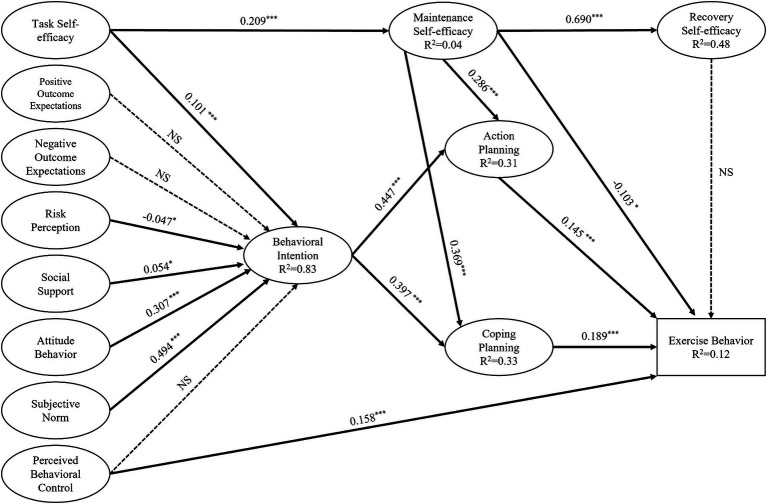
Standardized path coefficients for the structural equation model explaining and predicting exercise behavior in individuals with SUDs based on the HAPA-TPB integrated model (M1). Non-significant paths are denoted by NS; ^*^p < 0.05, ^**^p < 0.01, ^***^p < 0.001.

The integrated HAPA-TPB model was estimated and tested using the maximum likelihood method, the standardized path coefficients of each path in the model were checked, and the M_1_ model shown in Figure 1 was obtained as follows: *χ^2^*/*df* = 4.33, RMSEA = 0.053, NFI = 0.919, CFI = 0.937, IFI = 0.937; this model fit the data well. As shown in Figure 1, the integrated model strongly predicted behavioral intentions, explaining 83% of the variance in behavioral intentions, 31% of the variance in action planning, 33% of the variance in coping planning and 12% of the variance in exercise behavior. Task self-efficacy explained 4% of the variance in maintenance self-efficacy, and maintenance self-efficacy explained 48% of the variance in recovery self-efficacy. There was an increase in predictive power compared to that of the HAPA model alone (Shen et al., 2012).

The results of the path analysis of the latent variables in the integrated model are shown in Table 4. Social support (*p* < 0.05), behavioral attitudes (*p* < 0.001), subjective norms (*p* < 0.001), and task self-efficacy (*p* < 0.001) were positively correlated with behavioral intentions, whereas risk perceptions (*p* < 0.05) were negatively correlated with behavioral intentions. Regarding exercise behavior in individuals with SUD, action planning (*p* < 0.001), coping planning (*p* < 0.001), and perceived behavioral control (*p* < 0.01) were positively correlated with exercise behavior, while maintenance self-efficacy (*p* < 0.05) was negatively correlated with exercise behavior. Task self-efficacy (*p* < 0.001) and maintenance self-efficacy were positively correlated. Maintenance self-efficacy was positively correlated with recovery self-efficacy (*p* < 0.001), action planning (*p* < 0.01), and coping planning (*p* < 0.01). Behavioral intentions were positively correlated with action planning (*p* < 0.001) and coping planning (*p* < 0.001). The remaining paths were not significant (*p* > 0.05).

**Table 4:** Results of standardized path analysis.

Relationships	Standardized coefficient (β)	95% CI		*p*-values
		Lower	Upper	
Task self-efficacy → Maintenance self-efficacy	0.209	0.138	0.278	0.000
Positive outcome expectations → Behavioral intention	−0.048	−0.116	0.011	0.107
Negative outcome expectations → Behavioral intention	0.023	−0.017	0.075	0.221
Risk perception → Behavioral intention	−0.047	−0.092	−0.010	0.014
Social support → Behavioral intention	0.054	0.012	0.111	0.014
Attitude behavior → Behavioral intention	0.307	0.193	0.426	0.000
Subjective norm → Behavioral intention	0.494	0.379	0.619	0.000
Perceived behavioral control → Behavioral intention	0.100	−0.028	0.222	0.121
Task self-efficacy → Behavioral intention	0.101	0.051	0.169	0.000
Maintenance self-efficacy → Recovery self-efficacy	0.690	0.631	0.740	0.000
Behavioral intention → Action planning	0.447	0.393	0.516	0.000
Maintenance self-efficacy → Action planning	0.286	0.219	0.343	0.001
Behavioral intention → Coping planning	0.397	0.340	0.468	0.000
Maintenance self-efficacy → Coping planning	0.369	0.297	0.427	0.001
Action planning → Exercise behavior	0.145	0.062	0.227	0.000
Maintenance self-efficacy → Exercise behavior	−0.103	−0.199	−0.007	0.048
Recovery self-efficacy → Exercise behavior	−0.050	−0.153	0.058	0.368
Coping planning → Exercise behavior	0.189	0.095	0.282	0.000
Perceived behavioral control → Exercise behavior	0.158	0.093	0.216	0.001

### 3.5 Mediating effects

Further analysis of the mediating effects indicated that task self-efficacy could act on exercise behavior both through maintenance self-efficacy and planning (all *p* < 0.001) and through behavioral intentions and planning (all *p* < 0.001) and could also directly affect exercise behavior by influencing maintenance self-efficacy (*p* < 0.05). Risk perception, social support, attitudes, and subjective norms play a role in exercise behavior through behavioral intentions and planning, including action planning and coping planning (all *p* < 0.01). Maintenance self-efficacy, behavioral intention, and planning were identified as chain mediating variables of the integrated model and had a significant mediating effect; the values of each mediating effect and 95% confidence intervals are shown in Table 5.

**Table 5:** Mediating effects.

Path	Effects (β)	95% CI		*p-values*
		Lower	Upper	
Task self-efficacy → Maintenance self-efficacy → Exercise behavior	−0.024	−0.055	0.000	0.047
Task self-efficacy → Maintenance self-efficacy → Action planning → Exercise behavior	0.010	0.004	0.020	0.000
Task self-efficacy → Maintenance self-efficacy → Coping planning → Exercise behavior	0.017	0.008	0.031	0.000
Task self-efficacy → Behavioral intention → Action planning → Exercise behavior	0.008	0.003	0.018	0.000
Task self-efficacy → Behavioral intention → Coping planning → Exercise behavior	0.009	0.004	0.021	0.000
Risk perception → Behavioral intention → Action planning → Exercise behavior	−0.003	−0.009	−0.001	0.008
Risk perception → Behavioral intention → Coping planning → Exercise behavior	−0.004	−0.010	−0.001	0.007
Social support → Behavioral intention → Action planning → Exercise behavior	0.004	0.001	0.012	0.010
Social support → Behavioral intention → Coping planning → Exercise behavior	0.005	0.001	0.013	0.008
Attitude behavior → Behavioral intention → Action planning → Exercise behavior	0.020	0.009	0.038	0.000
Attitude behavior → Behavioral intention → Coping planning → Exercise behavior	0.024	0.011	0.044	0.000
Subjective norm → Behavioral intention → Action planning → Exercise behavior	0.039	0.019	0.069	0.000
Subjective norm → Behavioral intention → Coping planning → Exercise behavior	0.045	0.024	0.084	0.000
Perceived behavioral control → Behavioral intention → Action planning → Exercise behavior	0.008	0.000	0.021	0.061
Perceived behavioral control → Behavioral intention → Coping planning → Exercise behavior	0.007	0.000	0.020	0.064
Total indirect effects	0.174	0.125	0.248	0.000
Total effects	0.341	0.279	0.417	0.000

### 3.6 Impact of exogenous variables in the integrated model

**Figure 2:**
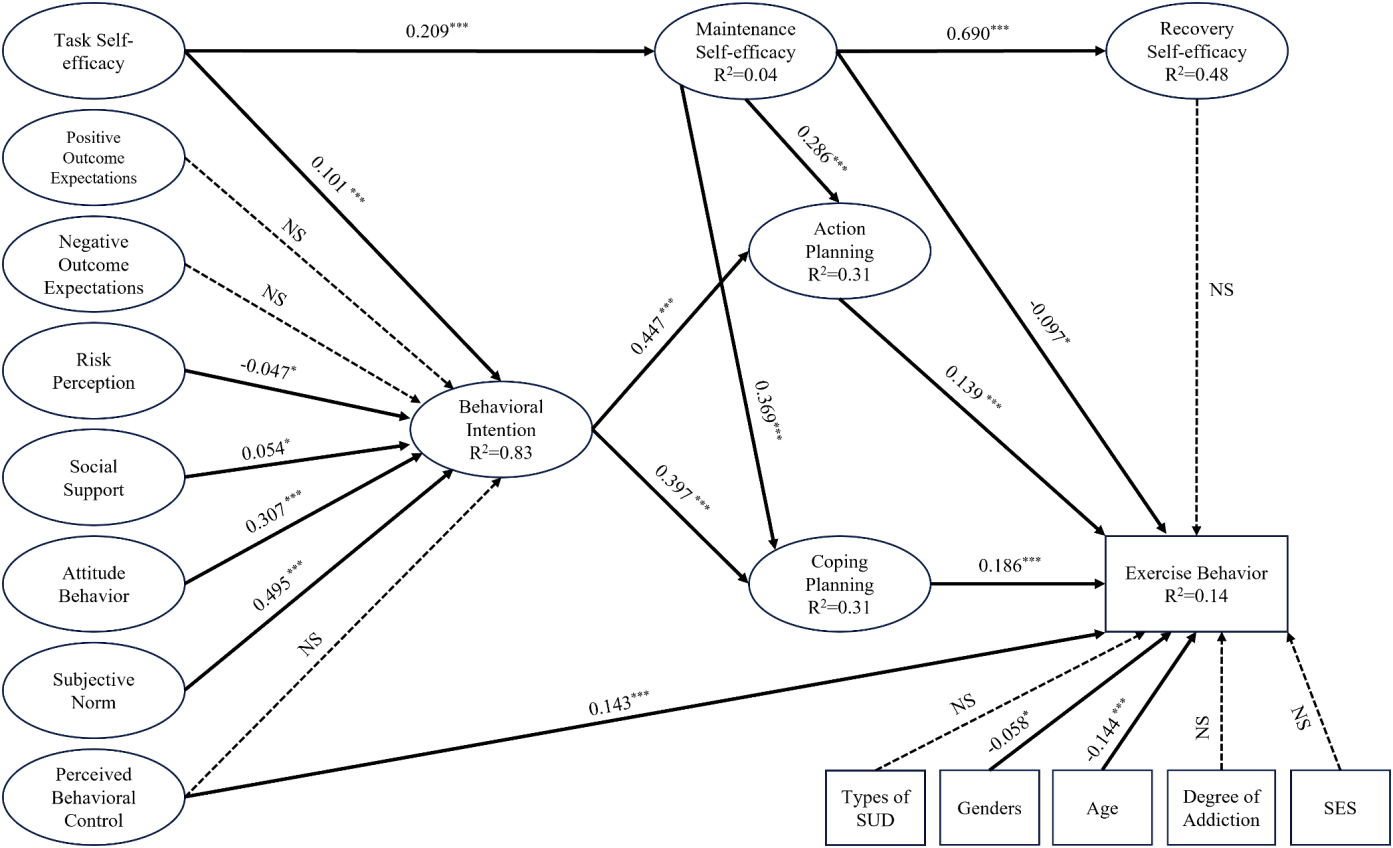
Standardized path coefficients of the exogenous variables in the HAPA-TPB integration model for explaining and predicting exercise behavior in individuals with SUDs (M2). Non-significant paths are denoted by NS; ^*^p < 0.05, ^**^p < 0.01, ^***^p < 0.001.

To further explore the possible effects of gender, age, type of addictive substance, degree of addiction and socioeconomic status on exercise behavior in individuals with SUD, this study further analyzed the integrated model by adding these five exogenous variables to the existing model (for details, see Model M2 in Figure 2). The results showed that for individuals with SUD, gender (*p* < 0.05) and age (*p* < 0.001) had significant negative effects on exercise behavior, while no direct effects of addiction level, addiction type, or socioeconomic status were found on exercise behavior. The various fit indices of M2 were *χ^2^/df* = 3.99, RMSEA = 0.050, NFI = 0.906, CFI = 0.928, and IFI = 0.928; this model had good fit.

## 4 Discussion

Physical exercise is considered a useful non-pharmacological intervention for promoting SUD recovery, and currently, low adherence rates weaken the rehabilitative benefits of physical exercise. Studies investigating the psychological correlates of the initiation and maintenance of physical exercise behavior in individuals with SUD are lacking. The purpose of this study was to test the feasibility and applicability of an integrated HAPA-TPB model for physical exercise behavior in individuals with SUD. The study revealed that the model can explain and predict the initiation and maintenance of physical exercise behavior in individuals with SUD.

The model includes the initiation and maintenance stages of physical exercise behavior in individuals with SUD. Regarding the initiation of physical exercise behavior, the results of the HAPA-TPB integration model indicated that task self-efficacy, risk perception, social support, behavioral attitudes, and subjective norms were indirectly associated with physical exercise behavior in individuals with SUD through behavioral intentions. In addition, perceived behavioral control was directly related to the physical exercise behavior of individuals with SUD. However, positive outcome expectancy, negative outcome expectancy, and perceived behavioral control did not significantly predict behavioral intention. In terms of task maintenance, the results indicated that maintenance self-efficacy, action planning, and coping planning were directly associated with physical exercise behavior in individuals with SUD. Moreover, maintenance self-efficacy was indirectly associated with physical exercise behavior in individuals with SUD through action planning and coping planning.

### 4.1 Variability analysis of different exercise phases

According to the HAPA-TPB model, the moderating variables of action self-efficacy, recovery self-efficacy, positive outcome expectancy, negative outcome expectancy, action planning, coping planning, social support, subjective norms, behavioral attitudes, behavioral intentions, perceived behavioral control, and exercise behaviors exhibited significant variability across the different phases of exercise behavior (as shown in Table 2). This finding implies that there is significant discontinuity in these moderating variables at different exercise behavior stages. Moreover, these findings also imply that these moderating variables play an important role in specific exercise phase transitions. Since discontinuity is an important basis for judging a model as a true model, it can be hypothesized that this integrated model is suitable for the prediction of physical exercise behavior in individuals with SUD.

### 4.2 Factors affecting intentions during the initiation stage

Neither outcome expectations (positive or negative) nor perceived behavioral control significantly predicted behavioral intentions during the behavioral initiation phase of the integrated HAPA-TPB model. These findings are inconsistent with those of several previous studies and theoretical hypotheses (Barg et al., 2012; Savvidou et al., 2012; Schwarzer, 2016; Wang et al., 2023). The possible reasons for this are as follows. First, individuals with SUD are required to complete physical exercise in a compulsory isolation setting in accordance with the established task schedule of rehabilitation institutions, so they are rarely able to exercise their judgment while controlling external conditions. Second, long-term drug abuse causes serious cardiorespiratory impairment in individuals with SUD (Kochetkova et al., 1998). Thus, during early physical exercise interventions, it is difficult for individuals with SUD to quickly experience the beneficial effects of physical exercise due to their physical weakness. Finally, in the context of the social stigmatization of drug addiction, it is difficult for individuals with SUD to gain social acceptance and recognition (Cazalis et al., 2023); as a result, it is more difficult for them to gain positive social appreciation through behavioral changes, such as the adoption of physical exercise habits. Excitingly, however, task self-efficacy, risk perception, social support, behavioral attitudes, and subjective norms significantly predicted behavioral intentions, explaining 83% of the variance, with subjective norms and behavioral attitudes being the top two predictors. This finding implies that the pressures from society and from compulsory isolation rehabilitation institutions regarding exercise behaviors demand that individuals with SUD experience prior to acting, as well as their combined assessment of the behavioral benefits of engaging in physical exercise, are the most important determinants of whether they can form behavioral intentions. Additionally, we observed that task self-efficacy and social support were significantly and positively related to behavioral intentions toward SUD. This implies that the beliefs of individuals with SUD in their own abilities and their social support from significant others (e.g., rehabilitation staff) are also important factors in forming their intentions before taking action. In addition, unexpectedly, we observed a significant negative correlation between risk perception and the behavioral intentions of individuals with SUD. This finding is inconsistent with previous findings on physical exercise behavior acquisition (Schwarzer et al., 2007) but reflects the previously identified strong relationship between risk perception and addictive substance use and withdrawal (Grevenstein et al., 2015). This finding suggested that individuals with SUD who perceive greater risk will have a decreased probability of quitting physical exercise to seek more rapid quitting substance use. Based on the above analysis of the factors influencing behavioral intention, we suggest the following. At the early stage of rehabilitation treatment in compulsory isolation drug rehabilitation institutions, it is necessary for rehabilitation staff, in collaboration with families of individuals with SUD, to increase their endorsement of and support for SUD participation in physical exercise. At the same time, it is also important to increase the knowledge dissemination and guidance of physical exercise for recovery. These strategies can effectively strengthen SUDs’ attitudes toward participating in physical exercise independently, increase their ability to develop higher self-efficacy, independently envision strategies to achieve desired outcomes, and prepare their intentions for the upcoming physical exercise.

### 4.3 Factors affecting exercise behavior during the maintenance stage

In the behavior maintenance stage in the integrated HAPA-TPB model, both behavioral intention and maintenance self-efficacy were significant predictors of planning, together explaining 32% of the variance. The current findings are consistent with a meta-analysis of studies applying the HAPA (Grevenstein et al., 2015). This finding implies that self-efficacy in coping with obstacles one may face when engaging in physical exercise and the intention to engage in that behavior are important factors in the development of a plan among individuals with SUD. Task self-efficacy also indirectly affects planning through behavioral intentions. This indirect effect has been addressed in past studies applying the HAPA (Schwarzer et al., 2007; Barg et al., 2012). Self-efficacy plays an important role in an individual’s behavioral change process. Individuals with SUD who have gone through the initiation phase of physical exercise behavior and then transitioned to the maintenance phase will encounter new challenges, and the implementation of self-efficacy interventions can help to improve the maintenance of physical exercise behavior among individuals with SUD. Therefore, we suggest that factors such as the self-efficacy of individuals with SUD and their exercise stage should be fully considered when developing physical exercise interventions so that they can be implemented more accurately to promote the maintenance of physical exercise behavior.

Maintenance self-efficacy and planning were found to be significant predictors of exercise behavior in individuals with SUD during the behavior maintenance phase of the integrated HAPA-TPB model. Consistent with the results of previous studies (Sniehotta et al., 2005a; Luszczynska and Sutton, 2006), our study revealed that maintenance self-efficacy was effective in predicting physical exercise behavior. Among people with SUD, a group that rarely participates in physical exercise prior to rehabilitation treatment through obligatory isolation, having self-efficacy to deal with the barriers that may arise when participating in physical exercise is essential for the maintenance of physical exercise. Taken together, the findings from the present study and the results of previous studies show that self-efficacy positively predicts behavioral intentions, planning, and physical exercise behavior. This result supports the theoretical assumptions of the HAPA-TPB integration model that we set out to develop. However, a positive predictive effect of recovery self-efficacy was not observed in our study, possibly because most of the individuals with SUD involved in this study were in the early stages of physical exercise, and fewer participants had experienced recovery self-efficacy.

### 4.4 Mediating role of action planning and coping planning

Numerous studies have shown that the gap between behavioral intentions and physical exercise behaviors is a barrier to the maintenance of physical exercise behaviors (Sniehotta et al., 2005a,b) and that programs play a critical role in bridging the gap between behavioral intentions and behaviors (Wee and Dillon, 2022). The role of programs in bridging the gap between behavioral intentions and behaviors is critical. Excitingly, planning was also observed in our study as a mediating variable in the behavioral intention → physical exercise behavior pathway, with planning being responsible for translating behavioral intention into sustained physical exercise behavior among people with SUD. Moreover, we also observed that planning was a mediating variable in the maintenance self-efficacy → physical exercise behavior pathway. To explore in detail the important role of plans in the integrated HAPA-TPB model, we subdivided plans into action planning and coping planning when constructing our theoretical model. In our study, we observed that behavioral intentions drove physical exercise behavior through action planning and coping planning. Through action planning, individuals with SUD have a mental representation of when and where to exercise and behavioral actions related to how to exercise when making physical exercise plans. This increases the effectiveness of physical exercise behavior change. Additionally, with coping planning, individuals with SUD anticipate potential barriers or disruptions (if-condition) and respond to difficulties that may interfere with the execution of physical exercise behavior (then-condition) (Patterson and Mischel, 1976). The contribution of coping planning is particularly important in a fixed and familiar mandatory segregated rehabilitation setting because individuals with SUD can anticipate potential barriers, which also makes it extremely easy to motivate them to make a psychological connection between foreseeable barriers and appropriate alternative planning, which increases the likelihood that they will continue to maintain physical exercise. In summary, it is clear that action planning and coping planning are important mediators of maintaining SUD physical exercise, a behavior that requires long-term maintenance and complex steps. Therefore, we suggest that in the process of rehabilitation treatment for individuals with SUD, especially in compulsory isolation rehabilitation situations, rehabilitation staff should help to formulate a feasible physical exercise plan, including the content, frequency, duration, and exercise schedule. And individuals with SUD need to be able to identify a stable place for physical exercise. Moreover, it is also necessary to help individuals with SUD identify stable places for exercise. Furthermore, it is important to guide individuals with SUD to independently construct coping strategies for various obstacles in the execution of physical exercise. Thus, the intention of individuals with SUD to develop action planning and their ability to cope with the difficulties of exercise behavior can be improved to promote the maintenance of physical exercise behavior.

### 4.5 Impact of exogenous variables

Our study also examined the effects of exogenous variables in the HAPA-TPB integration model. The results showed that only gender and age had significant effects on physical exercise behavior in individuals with SUD (*β* values of-0.058 and-0.144, respectively). Therefore, men with SUD are more likely to form physical exercise habits than women with SUD, although only a small difference exists. Moreover, for all individuals with SUD, the change in physical exercise behavior increasingly decreased with age. These findings are consistent with previous study findings (Hallal et al., 2012; Mao et al., 2020). Therefore, we suggest that during the development of physical exercise behavior in individuals with SUD, it is necessary for rehabilitation staff to provide more social support, more detailed physical exercise program development, and more specific physical exercise guidance to women with SUD and older individuals with SUD to promote the initiation and maintenance of physical exercise behavior in this group. In contrast, addiction type, addiction level, and socioeconomic status did not significantly affect physical exercise behavior in individuals with SUD. Previous studies have concluded that socioeconomic status has a significant effect on physical exercise behavior change (Ford et al., 1991; Cerin and Leslie, 2008; Welford et al., 2023), which was not observed in our study. This may be because the socioeconomic status indices of the respondents in our study were relatively concentrated (as shown in Table 1), and there was no significant difference between the high or low socioeconomic status groups. In addition, the type of addictive drug and the degree of addiction did not significantly affect the results of this integrated model. A possible reason for this difference is that stimulant drugs are more popular than heroin is, and 87.55% of the individuals with SUD in our survey were addicted to stimulant drugs or a polydrug of stimulant drugs or other drugs (as shown in Table 1); alcohol use disorders and tobacco use disorders were not included in our study. To explore the effects of drug addiction-related variables on physical exercise behavior in more detail, further expansion of the survey may be needed.

### 4.6 Strengths and limitations

This study has several strengths. First, the population studied was unique and indispensable. However, few studies have examined predictors of the initiation and maintenance of exercise behavior in individuals with SUD during physical exercise interventions. This group of individuals, which has become an important public health challenge worldwide, urgently needs to recognize and promote physical exercise based on relevant health behavior theories. Second, this study integrated two classical theories related to health behavior to investigate the determinants of physical exercise behavior in individuals with SUD by considering self-efficacy, behavioral intention, planning, and the exercise stage of physical exercise behavior. This is the first study to investigate the relationship between HAPA-TPB-related variables and physical exercise behavior in individuals with SUD. Therefore, our study is expected to stimulate additional research focusing on health behavior shaping in the population of people with SUD and to lay the foundation for future theoretical research and provide scientific guidance for rehabilitation practice.

Despite the above findings and strengths, there are several limitations of this study that should be considered. First, our participants were recruited from a compulsory drug rehabilitation center through convenience sampling, which may limit the generalizability of the findings to voluntary drug treatment SUD in community-oriented rehabilitation settings and medical rehabilitation facilities. Future research should investigate the factors influencing behavioral initiation and maintenance during physical exercise interventions in various voluntary drug treatment groups. Second, only 87 women with SUD (7%) were recruited for our survey sample. Although we observed a significant effect of gender on the HAPA-TPB score, this statistical bias may be due to the gender differences in the number of individuals with SUD, and gender was not included as a covariate in the model. Therefore, the sample of women with SUD should be increased in future studies to further validate the validity of the integrated HAPA-TPB model. Third, past exercise behavior or habitual intensity, as well as social cognitive factors, are thought to have direct or indirect effects on behavioral intentions and exercise behavior (Savvidou et al., 2012; Schwarzer, 2016). These factors can be included in future studies on the HAPA-TPB integration model. Fourth, the current study examined only the predictors of physical exercise behavior in individuals with SUD according to the HAPA-TPB integration model through a cross-sectional investigation; therefore, the results could not explain the dynamic effects of the variables of this integration model on chronic physical exercise behavior. Future longitudinal studies should be conducted to investigate the long-term effects of these variables on maintaining physical exercise behavior in individuals with SUD. Fifth, the indicators included in this integrative model were obtained through self-reports rather than direct observation, which may have triggered social approval bias and subjective bias. Therefore, psychological factors associated with SUD should be measured through more objective observation methods and psychological assessments in future studies. It is also expected that the physical exercise behavior of individuals with SUD could be measured by more objective tools, such as an actigraphy monitor based on the 3-axis accelerometer principle. Finally, although the HAPA-TPB integrates physical exercise behavior in individuals with SUD relatively well, the excellent performance of other theoretical models in predicting physical exercise behavior may provide new perspectives, such as self-determination theory (Hagger and Chatzisarantis, 2008; Teixeira et al., 2012) and temporal self-regulation theory, for the explanation of physical exercise behavior in individuals with SUD (Hall and Fong, 2007; Wang et al., 2023).

## 5 Conclusion

This study demonstrated that the integrated HAPA-TPB model has good applicability and validity for evaluating physical exercise behavior in individuals with SUD. In addition, the integrated model can effectively explain and predict the initiation and maintenance of physical exercise behavior in individuals with SUD. Task self-efficacy, risk perception, social support, behavioral attitudes, and subjective norms are important relational factors for the initiation of physical exercise behavior in individuals with SUD. Behavioral intention is an important mediating variable in the initiation of physical exercise behavior in individuals with SUD. Maintenance self-efficacy and perceived behavioral control are direct factors involved in maintaining physical exercise behavior in individuals with SUD. Action planning and coping planning are indirect factors involved in maintaining physical exercise behavior in individuals with SUD.

## Data availability statement

The original contributions presented in the study are included in the article/supplementary material, further inquiries can be directed to the corresponding authors.

## Ethics statement

The studies involving humans were approved by Ethics Review Board, Faculty of Sport and Science, Ningbo University. The studies were conducted in accordance with the local legislation and institutional requirements. The participants provided their written informed consent to participate in this study.

## Author contributions

YM: Writing – original draft, Conceptualization, Investigation, Methodology, Resources. TZ: Conceptualization, Methodology, Writing – original draft. WC: Writing – review & editing, Data curation, Formal analysis, Methodology. HZ: Writing – original draft, Data curation, Formal analysis, Investigation. LT: Data curation, Investigation, Writing – original draft. XWa: Data curation, Investigation, Writing – original draft. ML: Data curation, Investigation, Writing – original draft. XZ: Writing – original draft, Data curation, Investigation. DW: Conceptualization, Funding acquisition, Project administration, Writing – original draft, Writing – review & editing. XWu: Data curation, Investigation, Writing – original draft. SL: Investigation, Writing – review & editing, Data curation. CH: Investigation, Resources, Writing – review & editing.
